# The College of Nursing (Limited by Guarantee)

**Published:** 1920-11-06

**Authors:** 


### The College of Nursing
### (Limited by Guarantee).

BIRMINGHAM THREE COUNTIES CENTRE.

On Thursday, October 28, in the lecture theatre at the General Hospital, Birmingham (by kind permission of the Governors), Dr. D. K. Wilkinson gave an interesting address on "Some Diseases of the Heart." In the course of his lecture Dr. Wilkinson emphasised the need of rest and the value of massage and graduated exercise in training the heart to accommodate itself to changed physiological conditions, the recovery of the patient being largely dependent on the skill, tact, and efficiency of the nurse. The lecture was well attended and greatly appreciated.

EDINBURGH CENTRE.

The second annual general meeting of members will be held on Wednesday, November 10, at 3.30 P.M., in the Nurses' Club, 8 Drumsheugh Gardens. Miss Gill, R.R.C., will preside. A full attendance is requested.

